# Economic Burden of Major Diseases in China in 2013

**DOI:** 10.3389/fpubh.2021.649624

**Published:** 2021-05-19

**Authors:** Xianyan Song, Lan Lan, Ting Zhou, Jin Yin, Qiong Meng

**Affiliations:** ^1^Institute of Hospital Management, West China Hospital, Sichuan University, Chengdu, China; ^2^West China Biomedical Big Data Center, West China Hospital, Sichuan University, Chengdu, China; ^3^Department of Epidemiology and Biostatistics, West China School of Public Health and West China Fourth Hospital, Sichuan University, Chengdu, China; ^4^Department of Epidemiology and Health Statistics, School of Public Health, Kunming Medical University, Kunming, China

**Keywords:** direct economic burden, indirect economic burden, disease, China, decision making

## Abstract

Studies on the economic burden of disease (EBD) can estimate the social benefits of preventing or curing disease. The majority of studies focus on the economic burden of a single or regional disease; however, holistic or national research is rare in China. Estimating the national EBD can provide evidence for policy makers. We used the top-down method to assess the economic burden of 30 types of diseases between urban and rural areas in China. The two-step model was used to evaluate the direct economic burden of disease (DEBD), while the human capital method was used to assess the indirect economic burden of disease (IEBD). The total economic burden of 30 types of diseases in China was between $13.39 and 803.00 billion in 2013. The average total economic burden of disease (TEBD) in cities was $81.39 billion, while diseases in villages accounted for $50.26 billion. The range of direct and indirect EBD was $5.77–494.52 billion, and the range in urban areas was $0.61–20.34 billion. The direct and indirect EBD in rural areas accounted for $5.88–277.76 billion and $0.59–11.39 billion, respectively. There was a large difference between the economic burden of different diseases. The economic burden of urban diseases was more significant than the burden for the rural. The top five most economically burdensome diseases were myocardial infarction coronary artery bypass, acute myocardial infarction, cerebral hemorrhage, acute upper gastrointestinal bleeding and acute appendicitis.

## Introduction

The rapid development of the economy has improved living and nutritional standards. Simultaneously, it has had a large effect on changing the spectrum of diseases in China (1, 2). Chronic non-communicable diseases are currently the prevailing diseases, increasing number of people paying more attention to their health. Furthermore, the study of the economic burden of disease (EBD) can help estimate the social benefits, resulting in the prevention or cure of disease, and can provide information related to the economic evaluation of disease (3–6). More importantly, this type of research offers a scientific basis for limited medical insurance and a rational allocation of health resources.

China introduced a new medical system reform policy on March 17, 2009. The main purpose of the new policy was to solve the problems that made it difficult and expensive for patients to visit a doctor. The new policy has decreased the damage on patients' bodies and the loss to society caused by illnesses. However, if the main disease can be prevented by implementing certain intervening measures with limited health resources, injuries and losses for individuals, families and societies that are caused by the disease can be avoided. Due to China's unique economic development, there is great economic disparity between urban and rural areas in China, which has greatly affected the constituent of the disease and the treatment options that are available to residents living between urban and rural areas. The majority of researches on economic burden has mostly focused on a single disease or localized diseases (7–11), but an integrated study of multiple diseases is rare in China. Recent studies only estimated the economic burden of lung cancer (12), seasonal influenza (13), chronic diseases (14), children with asthma (15) and Alzheimer's disease (16) in China, and the economic burden of Alzheimer's disease in Zhejiang Province (17), hepatitis E-infected patients in Jiangsu Province (18) and rare diseases in Shanghai (19) etc. Therefore, we intend to estimate the overall EBD and then, to separately measure the EBD between urban and rural areas in China in order to provide evidence for relevant policy makers.

## Methods

We used a top-down approach to estimate EBD in China. The top-down approach (6) finds national or regional total health expenditures in advance, relying on the existing system for information on health expenditures. Then, the various expenses are classified, such as hospitalization costs, outpatient and emergency costs, drug costs, etc., based on a certain percentage corresponding to the allocation of items used to target disease. Lastly, the average cost of individual or various diseases is calculated.

### Data Collection

The Analysis Report of Notional Health Services Survey in China 2013 (ARNHSS) provided consultation rates data for 2 weeks, categorized by the diseases and hospitalization rates and constituent ratio of outpatient and inpatient institutions in 2013. The China Health and Family Planning Statistical Yearbook 2014 (CHFPSY) provided the data for the average outpatient costs, hospitalization costs and the length of stay by diseases at different hospital levels and the total national health expenditure in 2013. The China Statistical Yearbook 2014 (CSY) provided the urban and rural population numbers and the Gross Domestic Product (GDP) data in 2013.

Every 5 years, the Chinese government conducts the national health services survey, which is a special sample survey, in order to understand the urban and rural residents' health status, health care utilization, health care costs and burden on the country. In 2013, the fifth national health services survey adopted multi-stage stratified cluster random sampling. The sample covered 31 provinces, 156 counties, 780 towns and 1,560 villages. It collected data from a total of 93,600 families, which is ~30 million people and is a good representative sample of China. The data from CHFPSY and CSY were reported by the administrative agency that is highly credible and known for high quality work. Furthermore, in order to obtain a consistent statistical caliber, each analytical index in CHFPSY was as closely matched as possible with ARNHSS in China.

### Research Disease

We selected 30 types of diseases from CHFPSY for this study to estimate their direct and indirect economic burden because the classification of diseases did not precisely match between CHFPSY and ARNHSS in China. Additionally, the CHFPSY classification of diseases was coded according to ICD-10. Therefore, it can be deduced that data integration was in process. Firstly, we listed the 30 types of diseases from CHFPSY 2014, and then we matched the name of the disease with ARNHSS in China 2013. If we could not find the exact same name of the disease, we classified the diseases into related systems according to the systems of diseases or main symptoms. If they still did not match using this method, the disease was classified as missing.

### Direct Economic Burden of Disease (DEBD)

DEBD refers to the total direct costs for the prevention and treatment of diseases, including various costs incurred by the individual, family and society for preventing, diagnosing, treating and rehabilitating the diseases and injuries (20). On the one hand, it includes the costs of health services provided by the health institutions, such as the prevention of capital costs, emergency expenses, outpatient diagnosis and treatment costs, hospital expenses, medical expenses, health technology labor, family bed treatment and care costs, etc. On the other hand, other charges paid by the patients while receiving health services are included, such as nutrition fees, transportation fees, travel expenses, non-prescription costs for purchasing rehabilitation equipment, etc. We estimated the direct economic burden of 30 types of diseases, including outpatient medical expenses and inpatient medical expenses only because we don't have non-medical expenses. The formula is as follows:

*DEBDi* = *N* × [2-week consultation rate × 26 × (Σ outpatient facilities constituent ratio × average outpatient medical cost in outpatient institutions at different levels) + hospitalization rate × (Σ inpatient facilities constituent ratio × average hospitalization medical expense in inpatient institutions at different levels)] (1)

In the above formula, *DEBDi* is the direct economic burden for the class *i* disease, and *N* is the number of people in the research population.

### Indirect Economic Burden of Disease (IEBD)

IEBD refers to a society and family's loss between the current value and future potential value due to illness that may result in the reduction of effective working time and the ability to work (21). This is also known as indirect costs. Indirect costs represent the value given by society to health and life. While it broadly includes social productivity losses, losses of income, losses of housework, employment costs, training costs, insurance costs, management costs, etc., indirect costs indicate the loss of productivity in this narrow sense. We used the human capital approach (22) to estimate the indirect economic burden of 30 types of diseases and calculate the economic loss of missed working time due to hospitalization. The formula is as follows:

*IEBDi* = *N* × hospitalization rate × (Σ inpatient facilities constituent ratio × average hospitalization days in inpatient institutions at different levels) × GDP/365 (2)

In the above formula, *IEBDi* is the indirect economic burden for the class *i* disease, and *N* is the number of people in the research population.

## Results

### DEBD

We weighted the average outpatient medical costs that were not caused by disease according to the constituent ratio of first diagnosis mechanism for urban outpatients over 2 weeks. The proportions of average outpatient medical costs in tertiary, secondary and primary hospitals were 14.46, 18.18, and 67.36%, respectively. In 2013 for urban patients, there were 16.04% distributed in tertiary hospitals, 69.28% distributed in secondary hospitals, and 14.68% distributed in primary hospitals. This distribution was used to weigh the average medical costs of hospitalization. China had a population of 731.11 million urban residents in 2013. The result of the outpatient costs, hospitalization costs and DEBD in urban China are shown in Table 1, which is based on Formula (1). The largest DEBD in urban areas was myocardial infarction coronary artery bypass (MICAB), and the second largest was acute myocardial infarction (AMI), while the smallest DEBD was primary nephrotic syndrome (PNS).

**Table 1:** Direct economic burden of 30 types of diseases in urban areas in China in 2013.

**Name of disease**	**2-week consultation rate (%0)**	**Weighted average outpatient costs ($)**	**Outpatient costs ($billion)**	**Inpatient rate (%0)**	**Weighted average inpatient costs ($)**	**Inpatient costs ($billion)**	**Direct economic burden ($billion)**
Viral hepatitis	0.46	28.73	2.50	0.79	1,237.78	7.19	9.69
Infiltrative pulmonary tuberculosis	0.46	28.73	2.50	0.79	1,217.77	7.08	9.57
Acute myocardial infarction	3.37	28.73	18.39	8.07	2,871.28	169.33	187.72
Congestive heart failure	3.37	28.73	18.39	8.07	1,141.58	67.32	85.71
Bacterial pneumonia	0.51	28.73	2.78	2.77	805.43	16.29	19.07
Chronic pulmonary heart disease	3.37	28.73	18.39	8.07	1,278.13	75.38	93.76
Acute upper gastrointestinal bleeding	7.63	28.73	41.68	9.78	1,241.93	88.83	130.51
Primary nephrotic syndrome	0.34	28.73	1.84	0.53	1,010.48	3.93	5.77
Hyperthyroidism	8.85	28.73	48.32	4.74	865.29	29.97	78.29
Cerebral hemorrhage	2.26	28.73	12.32	7.06	2,475.15	127.79	140.12
Cerebral infarction	2.26	28.73	12.32	7.06	1,302.97	67.27	79.60
Aplastic anemia	0.34	28.73	1.84	0.53	1,130.33	4.40	6.24
Acute leukemia	0.34	28.73	1.84	0.53	2,131.53	8.30	10.14
Nodular goiter	0.34	28.73	1.84	1.98	1,422.72	20.59	22.43
Acute appendicitis	7.63	28.73	41.68	9.78	1,020.75	73.01	114.69
Acute cholecystitis	0.68	28.73	3.73	2.04	1,194.06	17.80	21.53
Inguinal hernia	1.48	28.73	8.11	1.24	947.41	8.57	16.67
Malignant gastric tumor	0.91	28.73	5.00	4.90	2,492.69	89.35	94.35
Pulmonary malignant tumor	0.91	28.73	5.00	4.90	1,807.28	64.78	69.78
Esophageal malignant tumor	0.91	28.73	5.00	4.90	2,406.22	86.25	91.25
Myocardial infarction coronary artery bypass	3.37	28.73	18.39	8.07	8,073.60	476.13	494.52
Malignant neoplasm of bladder	0.91	28.73	5.00	4.90	2,139.75	76.70	81.69
Benign prostatic hyperplasia	3.27	28.73	17.85	5.23	1,553.33	59.43	77.28
Intracranial injury	1.08	28.73	5.90	1.70	1,850.28	23.02	28.92
Prolapse of lumbar intervertebral disc	6.51	28.73	35.58	6.12	1,308.20	58.51	94.09
Bronchial pneumonia in children	3.37	28.73	18.39	2.77	432.84	8.75	27.14
Infectious diarrhea in children	3.37	28.73	18.39	9.78	269.22	19.26	37.64
Leiomyoma of uterus	3.37	28.73	18.39	1.24	1,428.34	12.92	31.30
Cesarean section	0.11	28.73	0.61	9.51	944.71	65.71	66.32
Senile cataract	0.73	28.73	4.01	2.00	907.82	13.28	17.30

Similar to the method used to process urban medical costs, we weighted the average rural outpatient medical costs. As a result, the proportions of average rural outpatient medical costs in tertiary, secondary and primary hospitals for rural outpatients were 2.53, 16.31, and 81.16% in 2013, respectively. In 2013, there were 13.11% of rural inpatients distributed in tertiary hospitals, 56.61% distributed in secondary hospitals, and 30.28% distributed in primary hospitals. China had a rural population of 629.61 million residents in 2013. The result of the outpatient costs, hospitalization costs and DEBD in rural China are shown in Table 2, which is based on Formula (1). The largest DEBD in rural areas was MICAB, and the second largest was acute upper gastrointestinal bleeding (AUGIB), while the smallest DEBD was PNS.

**Table 2:** Direct economic burden of 30 types of diseases in rural areas in China in 2013.

**Name of disease**	**2-week consultation rate (%0)**	**Weighted average outpatient costs ($)**	**Outpatient costs ($billion)**	**Inpatient rate (%0)**	**Weighted average inpatient costs ($)**	**Inpatient costs ($billion)**	**Direct economic burden ($billion)**
Viral hepatitis	0.43	25.75	1.80	1.25	1,088.56	8.55	10.35
Infiltrative pulmonary tuberculosis	0.43	25.75	1.80	1.25	993.99	7.81	9.61
Acute myocardial infarction	2.84	25.75	11.96	5.79	2,133.82	77.85	89.81
Congestive heart failure	2.84	25.75	11.96	5.79	1,005.15	36.67	48.63
Bacterial pneumonia	0.63	25.75	2.67	3.11	625.97	12.25	14.92
Chronic pulmonary heart disease	2.84	25.75	11.96	5.79	1,047.51	38.22	50.18
Acute upper gastrointestinal bleeding	9.59	25.75	40.42	10.55	1,031.80	68.53	108.95
Primary nephrotic syndrome	0.48	25.75	2.01	0.72	854.14	3.87	5.88
Hyperthyroidism	3.93	25.75	16.56	2.34	733.45	10.80	27.35
Cerebral hemorrhage	2.19	25.75	9.22	6.74	2,104.12	89.33	98.55
Cerebral infarction	2.19	25.75	9.22	6.74	1,063.90	45.17	54.39
Aplastic anemia	0.48	25.75	2.01	0.72	868.45	3.94	5.95
Acute leukemia	0.48	25.75	2.01	0.72	1,546.24	7.01	9.02
Nodular goiter	0.27	25.75	1.14	2.05	1,184.70	15.26	16.40
Acute appendicitis	9.59	25.75	40.42	10.55	862.14	57.26	97.68
Acute cholecystitis	0.88	25.75	3.73	1.86	934.11	10.94	14.67
Inguinal hernia	1.52	25.75	6.40	1.13	806.33	5.75	12.15
Malignant gastric tumor	0.61	25.75	2.55	2.98	1,862.85	34.94	37.50
Pulmonary malignant tumor	0.61	25.75	2.55	2.98	1,406.94	26.39	28.95
Esophageal malignant tumor	0.61	25.75	2.55	2.98	1,901.10	35.66	38.22
Myocardial infarction coronary artery bypass	2.84	25.75	11.96	5.79	7,285.04	265.80	277.76
Malignant neoplasm of bladder	0.61	25.75	2.55	2.98	1,744.04	32.72	35.27
Benign prostatic hyperplasia	3.12	25.75	13.16	5.58	1,335.90	46.94	60.10
Intracranial injury	0.96	25.75	4.06	1.47	1,480.87	13.69	17.75
Prolapse of lumbar intervertebral disc	7.83	25.75	33.02	5.83	934.17	34.29	67.32
Bronchial pneumonia in children	2.84	25.75	11.96	3.11	363.62	7.11	19.07
Infectious diarrhea in children	2.84	25.75	11.96	10.55	255.41	16.96	28.92
Leiomyoma of uterus	2.84	25.75	11.96	1.13	1,199.35	8.56	20.52
Cesarean section	0.08	25.75	0.33	10.02	810.88	51.16	51.50
Senile cataract	0.64	25.75	2.70	1.72	743.22	8.04	10.74

### IEBD

We weighted average hospitalization days by disease based on the constituent ratio for China's urban inpatients in 2013. The GDP per capita was $6,807 in China in 2013. The indirect economic losses of urban patients caused by hospitalization are shown in Table 3, which is based on Formula (2). The largest IEBD in urban areas was MICAB, which was followed by cerebral hemorrhage (CH), and the smallest IEBD was aplastic anemia (AA).

**Table 3:** Indirect economic burden of 30 types of diseases in urban areas in China in 2013.

**Name of disease**	**Inpatient rate (%0)**	**Weighted inpatient days (day)**	**Daily GDP per capita ($)**	**Indirect economic burden ($billion)**
Viral hepatitis	0.79	15.70	18.65	1.70
Infiltrative pulmonary tuberculosis	0.79	12.84	18.65	1.39
Acute myocardial infarction	8.07	9.08	18.65	9.99
Congestive heart failure	8.07	9.90	18.65	10.88
Bacterial pneumonia	2.77	8.78	18.65	3.31
Chronic pulmonary heart disease	8.07	11.11	18.65	12.22
Acute upper gastrointestinal bleeding	9.78	7.79	18.65	10.39
Primary nephrotic syndrome	0.53	12.12	18.65	0.88
Hyperthyroidism	4.74	8.89	18.65	5.75
Cerebral hemorrhage	7.06	14.93	18.65	14.38
Cerebral infarction	7.06	11.64	18.65	11.21
Aplastic anemia	0.53	8.36	18.65	0.61
Acute leukemia	0.53	14.91	18.65	1.08
Nodular goiter	1.98	8.19	18.65	2.21
Acute appendicitis	9.78	6.87	18.65	9.16
Acute cholecystitis	2.04	8.50	18.65	2.36
Inguinal hernia	1.24	6.94	18.65	1.17
Malignant gastric tumor	4.90	13.61	18.65	9.09
Pulmonary malignant tumor	4.90	14.11	18.65	9.43
Esophageal malignant tumor	4.90	15.79	18.65	10.55
Myocardial infarction coronary artery bypass	8.07	18.50	18.65	20.34
Malignant neoplasm of bladder	4.90	14.06	18.65	9.40
Benign prostatic hyperplasia	5.23	11.67	18.65	8.32
Intracranial injury	1.70	12.27	18.65	2.85
Prolapse of lumbar intervertebral disc	6.12	11.00	18.65	9.17
Bronchial pneumonia in children	2.77	6.88	18.65	2.60
Infectious diarrhea in children	9.78	4.94	18.65	6.59
Leiomyoma of uterus	1.24	9.44	18.65	1.59
Cesarean section	9.51	6.69	18.65	8.68
Senile cataract	2.00	4.26	18.65	1.16

Similar to the method used for dealing with urban patients' data, we weighted average hospitalization days by disease to obtain rural patients' hospitalization days by disease. The indirect economic losses of rural patients due to hospitalization are demonstrated in Table 4, which is based on Formula (2). The largest IEBD in rural areas was CH, which was followed by MICAB, and the smallest IEBD was AA.

**Table 4:** Indirect economic burden of 30 types of diseases of rural area in China, 2013.

**Name of disease**	**Inpatient rate (%0)**	**Weighted average inpatient days (day)**	**Daily GDP per capita ($)**	**Indirect economic burden ($billion)**
Viral hepatitis	1.25	15.58	18.65	2.28
Infiltrative pulmonary tuberculosis	1.25	12.50	18.65	1.83
Acute myocardial infarction	5.79	8.94	18.65	6.09
Congestive heart failure	5.79	9.86	18.65	6.71
Bacterial pneumonia	3.11	8.46	18.65	3.09
Chronic pulmonary heart disease	5.79	10.74	18.65	7.31
Acute upper gastrointestinal bleeding	10.55	7.61	18.65	9.42
Primary nephrotic syndrome	0.72	11.55	18.65	0.98
Hyperthyroidism	2.34	8.61	18.65	2.36
Cerebral hemorrhage	6.74	14.39	18.65	11.39
Cerebral infarction	6.74	11.16	18.65	8.83
Aplastic anemia	0.72	7.01	18.65	0.59
Acute leukemia	0.72	12.37	18.65	1.05
Nodular goiter	2.05	8.17	18.65	1.96
Acute appendicitis	10.55	6.95	18.65	8.60
Acute cholecystitis	1.86	8.03	18.65	1.75
Inguinal hernia	1.13	7.01	18.65	0.93
Malignant gastric tumor	2.98	13.03	18.65	4.56
Pulmonary malignant tumor	2.98	13.88	18.65	4.86
Esophageal malignant tumor	2.98	15.06	18.65	5.27
Myocardial infarction coronary artery bypass	5.79	15.25	18.65	10.38
Malignant neoplasm of bladder	2.98	13.74	18.65	4.81
Benign prostatic hyperplasia	5.58	11.27	18.65	7.39
Intracranial injury	1.47	11.54	18.65	1.99
Prolapse of lumbar intervertebral disc	5.83	10.43	18.65	7.14
Bronchial pneumonia in children	3.11	6.55	18.65	2.39
Infectious diarrhea in children	10.55	4.73	18.65	5.87
Leiomyoma of uterus	1.13	9.39	18.65	1.25
Cesarean section	10.02	6.62	18.65	7.79
Senile cataract	1.72	4.42	18.65	0.89

### Total Economic Burden of Disease (TEBD)

The total health expenditure of China in 2013 was $517.64 billion. The top five diseases among 30 types of diseases that had the heaviest TEBD based on the proportion in total health expenditure were MICAB (15.51%), AMI (5.67%), CH (5.11%), AUGIB (5.01%) and acute appendicitis (AAs) (4.45%). The last five diseases that had the smallest TEBD were AA (0.26%), PNS (0.26%), acute leukemia (AL) (0.41%), infiltrative pulmonary tuberculosis (IPT) (0.43%) and viral hepatitis (VH) (0.46%). The average TEBD for cities was $81.39 billion and for the villages was $50.26 billion, respectively. The total economic burden of 30 types of diseases in urban and rural areas in China is shown in Table 5.

**Table 5:** Total economic burden of 30 types of diseases in China in 2013.

**Name of disease**	**National**		**Urban**		**Rural**	
	**Total economic burden ($billion)**	**Proportion in total health expenses (%)**	**Total economic burden ($billion)**	**Proportion in total health expenses (%)**	**Total economic burden ($billion)**	**Proportion in total health expenses (%)**
Viral hepatitis	24.02	0.46	11.39	0.22	12.63	0.24
Infiltrative pulmonary tuberculosis	22.40	0.43	10.96	0.21	11.44	0.22
Acute myocardial infarction	293.61	5.67	197.71	3.82	95.90	1.85
Congestive heart failure	151.93	2.94	96.59	1.87	55.34	1.07
Bacterial pneumonia	40.39	0.78	22.38	0.43	18.01	0.35
Chronic pulmonary heart disease	163.47	3.16	105.98	2.05	57.49	1.11
Acute upper gastrointestinal bleeding	259.28	5.01	140.91	2.72	118.37	2.29
Primary nephrotic syndrome	13.52	0.26	6.65	0.13	6.86	0.13
Hyperthyroidism	113.75	2.20	84.04	1.62	29.72	0.57
Cerebral hemorrhage	264.44	5.11	154.5	2.98	109.95	2.12
Cerebral infarction	154.03	2.98	90.81	1.75	63.23	1.22
Aplastic anemia	13.39	0.26	6.85	0.13	6.54	0.13
Acute leukemia	21.29	0.41	11.22	0.22	10.07	0.19
Nodular goiter	43.00	0.83	24.64	0.48	18.36	0.35
Acute appendicitis	230.14	4.45	123.85	2.39	106.28	2.05
Acute cholecystitis	40.31	0.78	23.89	0.46	16.42	0.32
Inguinal hernia	30.93	0.60	17.85	0.34	13.09	0.25
Malignant gastric tumor	145.5	2.81	103.44	2.00	42.06	0.81
Pulmonary malignant tumor	113.01	2.18	79.21	1.53	33.80	0.65
Esophageal malignant tumor	145.28	2.81	101.80	1.97	43.48	0.84
Myocardial infarction coronary artery bypass	803.00	15.51	514.86	9.95	288.14	5.57
Malignant neoplasm of bladder	131.17	2.53	91.10	1.76	40.08	0.77
Benign prostatic hyperplasia	153.09	2.96	85.60	1.65	67.49	1.30
Intracranial injury	51.50	0.99	31.76	0.61	19.74	0.38
Prolapse of lumbar intervertebral disc	177.72	3.43	103.26	1.99	74.46	1.44
Bronchial pneumonia in children	51.20	0.99	29.74	0.57	21.46	0.41
Infectious diarrhea in children	79.02	1.53	44.23	0.85	34.79	0.67
Leiomyoma of uterus	54.66	1.06	32.89	0.64	21.77	0.42
Cesarean section	134.29	2.59	75.00	1.45	59.29	1.15
Senile cataract	30.09	0.58	18.46	0.36	11.63	0.22

## Discussion

This study integrally estimated the EBD of China. The TEBD was between $13.39 and 803.00 billion, which included 30 types of common diseases in China. This accounted for 2.54% of the average proportion of the national total health expenses; although, the highest proportion was 15.51%. This proportion is enough to capture our government's attention. The DEBD of cities was $5.77–494.52 billion, while the DEBD of villages was $5.88–277.76 billion. The IEBD for citizens was $0.61–20.34 billion, and the IEBD for the rural population was $0.59–11.39 billion. Whether in cities or countryside, the DEBD was much higher than the IEBD.

The DEBD, IEBD, and TEBD of VH, IPT, and PNS in urban areas were slightly lower than those in rural areas; however, the residual EBD of 27 types of diseases for the cities were higher than those for the countryside. Interestingly, the largest difference for the EBD of MICAB between urban and rural areas, which exceeded $226.72 billion for TEBD, was a relatively large difference for DEBD but a slight difference for IEBD. There is a possible explanation for this difference. One possible reason could be that there were more MICAB patients in cities than in the countryside because rural patients failed to visit a doctor because they were worried about incurring expensive medical bills.

The EBD had a large difference among different diseases. The average total economic burden of 30 types of diseases was $131.65 billion in China. The largest five diseases for TEBD were MICAB, AMI, CH, AUGIB, and AAs. The smallest five diseases for TEBD were AA, PNS, AL, IPT, and VH. The middle position for TEBD was held by malignant tumors.

It is important to note that this study has some limitations. The following costs or intangible economic burdens were not taken into consideration because of limited data: self purchased medical fees, the cost of time in bed, and cost of recovery time at home. Additionally, we could not measure the production value of the loss of life due to premature death and the loss of production value due to long-term disability caused by illness or disability, which will likely result in underestimating the EBD. The misclassification of the diseases may also result in errors.

## Conclusion

The EBD reflects the burden caused by illness for a society. If we can reduce or eliminate EBD, socioeconomic losses will decrease, and our society will benefit. However, policy makers must make allocations with limited resources. Therefore, this paper provides solid data and research for policy makers to make informed decisions. Overall, there was a large difference in the economic burden of different diseases, and the total economic burden of urban patients' disease was larger than that of rural patients. The top ten diseases were myocardial infarction coronary artery bypass, acute myocardial infarction, cerebral hemorrhage, acute upper gastrointestinal bleeding, acute appendicitis, prolapse of lumbar intervertebral disc, chronic pulmonary heart disease, cerebral infarction, benign prostatic hyperplasia and congestive heart failure.

## Data Availability Statement

Publicly available datasets were analyzed in this study. This data can be found here: http://www.stats.gov.cn/, http://www.nhc.gov.cn/.

## Author Contributions

LL and QM contributed to the design of the study and project management and contributed to the final interpretation of data. XS was the lead qualitative researcher and designed the process evaluation. QM developed the coding framework with LL and TZ. Coding and initial data interpretation was performed by LL, TZ, and JY. The first draft of the manuscript was produced by XS and LL. All authors critically reviewed and edited the draft paper, read and approved the final manuscript.

## Conflict of Interest

The authors declare that the research was conducted in the absence of any commercial or financial relationships that could be construed as a potential conflict of interest.
